# Statistics of Cases of Tetanus Observed in the Army Zones from November 1, 1915, to February 1, 1917

**Published:** 1918-01

**Authors:** 


***Statistics of Cases of Tetanus Observed in the Army Zones from November 1, 1915, to February 1, 1917.*** By M. CHAVASSE, Correspondant national. Trans. and Abs. from the *Bull. et Mém. de la Soc. de Chir. de Paris,* June 12, 1917.

C. bases his report upon 213 cases : 29 caused by accidents or surgical interventions; 38 by freezing; and 146 by wounds from projectiles. The observations are set forth with considerable detail.

C. thinks the intrathecal method of administration, efficatious but often impracticable because of the muscle contraction along the spinal column. He thinks that the intravenous method of injection of large doses of from 60 to 100 cc. was not sufficiently employed in the cases he observed.

He thinks there is no doubt that preventive injections of serum have more than proved their value, although it must be admitted that they have by no means prevented the occurrence of tetanus.

In general the gravity of the cases of tetanus was proportionate to the gravity of the wounds. Tetanus was especially severe in cases of trench feet. Its severity seemed to be diminished according to the number of preventive injections administered, even when very serious wounds were involved.

C. therefore urges that according to the severity of the wounds the usual preventive dose of 10 cc. be increased to 20 or 30 cc. and repeated at intervals of 7 or 8 days.

C. also supports the recommendation of Berard and Lumière that preventive doses should be administered before *all* operations. He cites 2 cases in which tetanus followed operations upon men for causes other than war wounds.

